# Y-shaped ZnO Nanobelts Driven from Twinned Dislocations

**DOI:** 10.1038/srep22494

**Published:** 2016-03-02

**Authors:** Yuan Shi, Shuhan Bao, Run Shi, Chengzi Huang, Abbas Amini, Zefei Wu, Linfei Zhang, Ning Wang, Chun Cheng

**Affiliations:** 1 Department of Materials Science and Engineering and Shenzhen Key Laboratory of Nanoimprint Technology, South University of Science and Technology, Shenzhen 518055, China; 2 Institute for Infrastructure Engineering, Western Sydney University, Kingswood, NSW 2751, Australia; 3 Department of Physics, Hong Kong University of Science and Technology, Hong Kong, China

## Abstract

Y-shaped ZnO nanobelts are fabricated by a simple thermal evaporation method. Transmission Electron Microscopy (TEM) investigation shows that these ZnO nanobelts are crystals with twinned planes {11–21}. Convergent Beam Electron Diffraction studies show that the two sides of twinned nanobelts are O-terminated towards the twinned boundary and Zn-terminated outwards. The two branches of twinned ZnO nanobelts grow along [11–26] from the trunk and then turn to the polarization direction [0001]. The featured Y-shape morphology and TEM characterizations indicate that the growth of these novel nanostructures is driven by an unusual twinned dislocation growth mechanism.

Zinc oxide with polar surfaces such as {0001}, from Zn- or O- terminated atomic planes, has shown to be an excellent candidate for novel and complicated nanostructures^1^,^2^,^3^,^4^,^5^,^6^,^7^,^8^,^9^,^10^,^11^,^12^,^13^,^14^,^15^,^16^,^17^,^18^,^19^,^20^,^21^,^22^,^23^,^24^,^25^,^26^,^27^,^28^,^29^. To date, numerous ZnO nanoscale morphologies such as nanowires^1^,^3^,^11^, nanoribbons^2^,^4^,^14^, nanobrushes^12^, etc., have been reported. Besides the intrinsic polarity characteristic of ZnO, structural defects such as heterophases^1^,^10^,^20^, screw dislocations^17^, twins^1^,^2^,^3^,^4^,^5^,^6^, etc., can effectively “engineer” the morphology of ZnO nanostructures. Amongst these, the twinned setup to connect building blocks can be an effective method for manually organizing nanostructures^1^,^2^,^3^,^4^,^5^,^6^. The introduction of twinned boundaries into ZnO nanostructures can strongly affect the electronic, magnetic, optical and mechanical properties of ZnO^7^,^8^,^9^,^10^. In general, the growth of these nanostructures opens an option for assembling nanoscale blocks into two-dimensional structures, and therein, the newly developed bicrystal ZnO nanostructures can significantly improve the development of novel functional devices.

The reflected twinned crystals of ZnO nanobelts have been reported more often than other similar bicrystal nanostructures. According to our investigation, there are two main kinds of reflected twinned ZnO nanocrystals: 1) the twinned planes are {1–10X} with X = 0, 1, 2, 3, 4 3,4,7,8,9; and 2) the twinned planes are {11–2Y} with Y = 2 2. In this work, we study a new type of Y-shaped twinned ZnO nanobelt with twinned planes {11–21} using transmission electron microscopy (TEM). Due to the importance of surface polarities on the growth morphology, we perform convergent-beam electron diffraction (CBED) experiments and simulations to determine the polarities of the nanostructures. The photoluminescence (PL) study shows that there is only one broad green emission which indicates a high density of point defects induced by twins.

## Results

**Figure 1:**
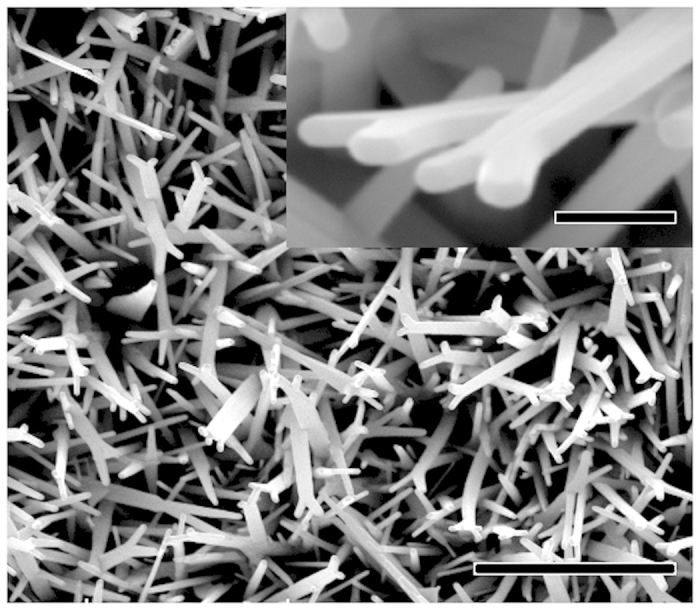
A SEM image of the as-synthesized twinned ZnO nanobelts. The inset is an enlarged image of the twinned ZnO nanobelts. Scale bar 10 μm and 1 μm for the inset.

Figure 1 shows the SEM image of as-synthesized products with a Y-shaped morphology and widths of several hundred of nanometers and lengths up to 10 micrometers. The high magnification SEM image in the inset of Fig. 1 clearly reveals that each Y-shaped structure consists of two separate nanobelts separated by a distinct grain boundary with the direction parallel to the growth direction, indicating the twinned crystal structures of the products. Unlike previously reported twinned ZnO nanostructures, the growth directions of these nanobelts change at the end of the twinned structure forming two branches.

**Figure 2:**
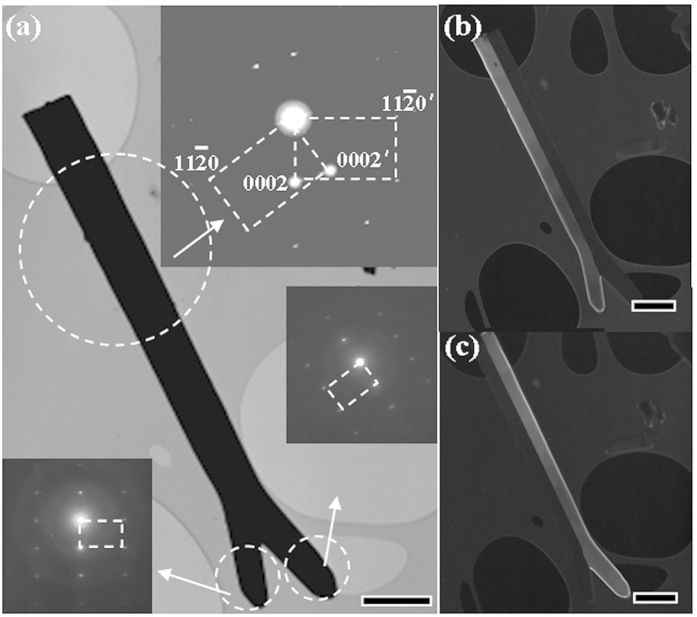
(**a**) Bright-field TEM image of a single twinned ZnO nanobelt. (**b**,**c**) Dark-field TEM images of twinned ZnO nanobelts recorded by a center objective aperture in the positions of (0001) and (0001’) of the SAED pattern taken from the whole twinned ZnO nanobelts respectively. The insets in (**a**) are SAED patterns recorded from the place as marked by arrows. Scale bar 1 μm for all.

TEM images demonstrate that the products are twinned ZnO nanobelts with a WZ structure. The twinned boundaries cannot be observed in the bright-field TEM image of the sample in Fig. 2a because of the quite large thickness of the sample, but, by using a higher accelerated voltage of 200 kV, these boundaries can be observed clearly. Selected Area Electron Diffraction (SAED) patterns taken from the trunk (inset Fig. 2a, as marked by an arrow) have two sets of spots with the [1–100] zone axis, which are the same as those taken from the two branches. This result reveals that the crystals on each branch follow the same orientation as the closest trunk part. Figure 2b,c show the dark field TEM images of the whole twinned ZnO nanobelt by using the central objective aperture in the (0001) and (0001’) positions of the SAED pattern. The diffraction contrast from the (0001) position gives the left part of the twinned nanobelts only while the diffraction contrast from the (0001’) position gives the right part of the twinned nanobelts. The smooth feature from the trunk to the branch in Fig. 2b,c reveals no stacking defects and coherent crystal orientations of each side of the twinned nanobelts. From the TEM observation in the dark field mode, it is revealed that the brightness of the nanobelt decreases from the trunk part to the branch part (Fig. 2b,c). This can be attributed to the decrease of the thickness of nanobelts during the growth stage.

**Figure 3:**
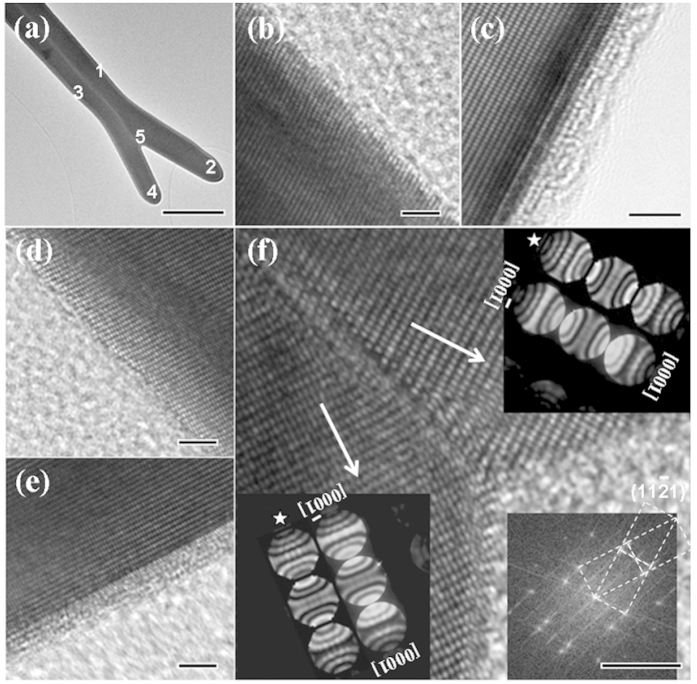
(**a**) Bright-field TEM images of a single twinned ZnO nanobelt for HRTEM. (**b**–**f**) are HRTEM images recorded from the position 1–5. The insets pointed by arrows in (**f**) are CBED patterns recorded at the two sides respectively and the simulated ones that are marked with stars. The insets at the right bottom in (**f**) are the corresponding Fast Fourier Transform (FFT) of (**f**). Scale bar 1 μm for (**a**); 5 μm for (**b**–**f**).

The structures of twinned nanobelts were further investigated using HRTEM and CBED. HRTEM images taken from different positions of a twinned ZnO nanobelt are shown in Fig. 3b–f. Figure 3f shows two sets of clear lattice images of [1–100] zone axis with a clear twinned boundary and the angle between the (0001) planes in the two crystals is 33°. The growth direction of the twinned nanobelt is [11–26], which is perpendicular to the [1–100] zone axis and in the {11–21} twinned plane (see the inset Fig. 3f at the right bottom). (11–21), [11–26] is a new twinned system other than those results previously reported. It is interesting that the growth directions of the two crystals change from [11–26] (trunk, Fig. 3b,d recorded from 1 and 3) to [0001] (branch, Fig. 3c,e recorded from 2 and 4). This special growth phenomenon can be correlated with the ZnO [0001] polar direction dominated growth^11^,^12^,^13^. To confirm this, a CBED study is carried out. The CBED patterns are formed with a converged electron probe focusing at the sample area in the nanometer range (for details, see ref. 11). Two CBED patterns recorded from the branches of the twinned nanobelts are shown in the insets of Fig. 3f (marked with arrows) along the [1–100] zone axis. The thickness of the twinned nanobelts is about 100 ~ 300 nm, which is appropriate for the CBED study. These experimental CBED patterns match fairly well with the simulated patterns (marked with stars, produced using JEMS simulation software)^14^,^15^. The best match was found for the sample with the thickness near to 120 nm, while the electron beam energy was set at 120 kV. We investigated a number of twinned ZnO nanobelts by using CBED and obtained the same result; namely that, the two sides of the twinned nanobelts are O-terminated towards the twinned boundary and Zn-terminated outwards.

## Discussion

Based on the growth conditions and observed structures, we believe the formation of the twinned ZnO nanobelts is as follows: first, the ZnO powders are vaporized and decomposed to Zn, ZnO_x_ and O_2_ at a high temperature and low pressure^11^. They are then transported to the lower temperature zone, where they later oxidize to ZnO nuclei. Then, the twin-like ZnO nuclei might be induced by a sudden change in the ambient pressure (increased from 1 Torr to 100 Torr) during the growth process, as previously reported^16^. It has been shown that the preferred condensations of metal vapor on the grain boundaries resulting in atomic steps along the twining and polar surfaces of an ionic crystal, make these positions as the fast growth sites^13^. Hence, we believe the final Y-shaped morphologies of twinned nanobelts are as a result of growth competition among these activated sites^17^. The presence of these twin-like ZnO nuclei can result in the fast growth in the [11–26] direction which is parallel to the twin plane and, finally, in the trunk formation of the twinned ZnO nanobelts. Afterwards, the growth in the [0001] direction dominates because of the changes in the growth condition (the decrease in temperature as well as the zinc vapor concentration) and, finally, the Y-shaped twinned nanobelts are obtained.

In application, the obtained Y-shaped twinned nanobelts structure with longer branches can be a modern candidate for electrical wiring, antenna, sensor, etc. Our experiments show that the length of the branch is not sensitive to the growth time. This may be because the growth condition window for branches is rather narrow. Further in-depth and comprehensive study is required for the evolution of these unique nanostructures.

**Figure 4:**
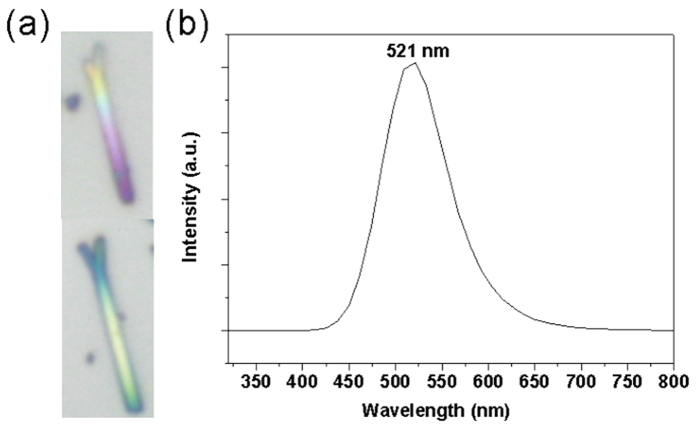
(**a**) High magnified optical images of single Y-shaped ZnO nanobelts. (**b**) The large area PL spectrum of Y-shaped ZnO nanobelts.

It is worthy to note that the two sides of the twinned nanobelts are O-terminated towards the twinned boundary and Zn-terminated outwards. This configuration can be attributed to the fact that the Zn-terminated surface is catalytically active and, thus, acts as the fast growth front. Almost in all ZnO nanostructures, Zn-terminated surface is outwards, such as in tetrapods^1^,^20^, nanobelts^2^,^4^,^14^, nanobrushes etc. Similar polarity induced during the growth stage has also been reported frequently in other AB types of wurtzite nanostructures, such as GaAs^30^,^31^, ZnSe^32^, GaN^33^,^34^ etc. Furthermore, the above noted growth is most likely to be controlled by a self-catalyzed VLS mechanism as no additional purities are added^11^,^18^. Large amounts of gray Zn powders are found in the temperature zone of less than 400 °C, indicating a rich zinc reactive atmosphere or a reactive atmosphere with the lack of oxygen, which further supports the self-catalyzed VLS growth. Since the thickness of as-synthesized twinned ZnO nanobelts ranges from 100 nm to 300 nm, right half of the light wavelength from ultra UV to yellow, the optical images (Fig. 4a) recorded from the samples that were dispersed on silicon wafer show gradual colors, which are caused by equal thickness interference. The different colors reveal an uneven thickness distribution, which is consistent with the result observed from the TEM images with the dark field mode.

PL spectra of ZnO nanobelts at room temperature are shown in Fig. 4b. A typical green defect emission at 521 nm is observed as commonly reported about ZnO nanostructures. This green emission can be attributed to the point defect O_zn_, that is, O^2−^ ion at the Zn^2+^ site^19^,^20^,^21^. It is interesting that no UV emission is observed. A previous work shows that only a weak UV emission appears when there exists a large number of twins in ZnO nanostructures^3^. For a comparison, ZnO nanowires grown with similar fabrication process without the sudden change in pressure are free of twin defects and they show a strong band-edge emission near 380 nm^11^. We believe the twinned structure of as-synthesized ZnO nanobelts can strongly inhibit the UV emission and the introduction of twin can be used as an effective method to tune the optical properties of ZnO nanostructures^20^.

In conclusion, we have demonstrated the fabrication of ZnO nanobelts with novel Y-shaped morphology through the thermal evaporation. The TEM investigation shows that the new type of ZnO nanobelts is created as twinned crystals with twinned planes {11–21}. CBED studies show that the two sides of the twinned nanobelts are O-terminated towards the twinned boundary and Zn-terminated outwards. The two branches of the twinned ZnO nanobelts grow along [11–26] from the trunk and then turn to the polarization direction [0001]. The photoluminescence (PL) study shows that there is only one broad green emission, indicating a high density of point defects induced by the novel twinned structure. Our strategy of controlled growth of ZnO Y-shaped nanostructures by the combination of polar direction and twin dislocation promisingly inspires a new way to tailor ZnO nanostructures for the design of novel functional devices^28^ for solar cells^22^,^23^, nanogenerators^24^, sensors^10^,^25^, electrical wiring^26^ and antenna^27^. A further study is in progress to introduce a pathway towards Y-shaped twinned ZnO nanobelts with prolonged branches.

## Methods

The Y-shaped twinned ZnO nanobelts were fabricated using the thermal evaporation method. In brief, high purity ZnO powder (99.9%) was placed at the center of an evacuated (1 ~ 2 Torr) tube furnace. A polycrystalline sapphire substrate was placed downstream in a lower temperature region (400–800 °C) of the furnace. After the furnace was heated to 1350 °C and held for 0.5 h^11^, the pressure was increased to 100 Torr within 1 minute for another 0.5 h. The temperature of system was raised to a designated set point at a rate of 10 °C min^−1^. An argon carrier gas was sent through the system at a rate of 25 sccm. After the reaction, the system cooled to room temperature and white products were found deposited on the substrate. Structural characterizations of the as-synthesized materials were characterized and analyzed by a scanning electron microscope (SEM, Philips XL30), and a JOEL JEM-2010F HRTEM equipped with EDX. The CBED patterns were recorded by using a Philips transmission electron microscope (CM120), and the CBED simulation was performed by using a JEMS simulation software. Optical images were recorded by an optical microscope (Olympus BX60). A room temperature large area PL was excited by the 325 nm line of He-Cd laser.

## Additional Information

**How to cite this article**: Shi, Y. *et al.* Y-shaped ZnO Nanobelts Driven from Twinned Dislocations. *Sci. Rep.* **6**, 22494; doi: 10.1038/srep22494 (2016).
